# The Role of Intercellular Signaling in the Regulation of Bacterial Adaptive Proliferation

**DOI:** 10.3390/ijms24087266

**Published:** 2023-04-14

**Authors:** Olga Petrova, Olga Parfirova, Natalia Gogoleva, Vladimir Vorob’ev, Yuri Gogolev, Vladimir Gorshkov

**Affiliations:** 1 Kazan Institute of Biochemistry and Biophysics, Federal Research Center “Kazan Scientific Center of the Russian Academy of Sciences”, 420111 Kazan, Tatarstan, Russia; 2 Institute of Fundamental Medicine and Biology, Kazan Federal University, 420008 Kazan, Tatarstan, Russia

**Keywords:** adaptive proliferation, intercellular communication, starvation stress, autoinducers, *Pectobacterium atrosepticum*, quorum sensing, cross-protection

## Abstract

Bacterial adaptation is regulated at the population level with the involvement of intercellular communication (quorum sensing). When the population density is insufficient for adaptation under starvation, bacteria can adjust it to a quorum level through cell divisions at the expense of endogenous resources. This phenomenon has been described for the phytopathogenic bacterium *Pectobacterium atrosepticum* (*Pba*), and it is called, in our study, adaptive proliferation. An important attribute of adaptive proliferation is its timely termination, which is necessary to prevent the waste of endogenous resources when the required level of population density is achieved. However, metabolites that provide the termination of adaptive proliferation remained unidentified. We tested the hypothesis of whether quorum sensing-related autoinducers prime the termination of adaptive proliferation and assessed whether adaptive proliferation is a common phenomenon in the bacterial world. We showed that both known *Pba* quorum sensing-related autoinducers act synergistically and mutually compensatory to provide the timely termination of adaptive proliferation and formation of cross-protection. We also demonstrated that adaptive proliferation is implemented by bacteria of many genera and that bacteria with similar quorum sensing-related autoinducers have similar signaling backgrounds that prime the termination of adaptive proliferation, enabling the collaborative regulation of this adaptive program in multispecies communities.

## 1. Introduction

Adaptive programs in bacteria are regulated at the population level, involving intercellular signaling [1,2,3,4,5]. Without intercellular communication, bacteria cannot properly adapt following exposure to a stressor. However, by the example of the phytopathogenic bacterium *Pectobacterium atrosepticum* (*Pba*), it has been shown that, if the cell concentration is below the one that permits intercellular communication, bacteria are able to increase the population density up to a quorum level, even in the absence of an exogenous growth substrate [6]. Such a phenomenon of an increase in population density under starvation conditions takes place only at a low initial cell titer of 10^1^–10^5^ CFU/mL until it reaches the value of ~10^6^ CFU/mL. In our study, we refer to this phenomenon as adaptive proliferation. Following adaptive proliferation, *Pba* cells retain high virulence and develop cross-protection, i.e., become resistant to a number of stressors [7].

Importantly, in the absence of exogenous organic carbon, irrespective of whether the initial cell titer is 10^1^ or 10^5^ CFU/mL, the adaptive proliferation yields a population density of ~10^6^ CFU/mL. This means that the adaptive proliferation is terminated not after a particular number of cell divisions, but after the population density reaches a particular level that presumably provides intercellular communication. This is supported by the facts that (1) the termination of the adaptive proliferation is coupled with the accumulation of autoinducers of one of the quorum sensing systems, acyl homoserine lactones (AHL), as well as with the upregulation of AHL-synthase gene, and (2) adaptive proliferation is terminated prematurely (at a level below 10^6^ CFU/mL) in the presence of metabolites accumulated in the starving cultures that had already passed through the process of adaptive proliferation [6].

Apparently, adaptive proliferation is carried out at the expense of endogenous cell resources, which is evident from cell morphology: cells after adaptive proliferation have reduced sizes and a dramatically reduced volume of cytoplasm [6]. Therefore, to prevent the waste of resources, it is important for bacterial cells to timely terminate adaptive proliferation when it is no longer needed, i.e., when the population density reaches a level enabling the intercellular communication necessary for an effective adaptation. It is obvious that cells “feel” this level via extracellular signaling molecules that remain unidentified to date. Quorum sensing autoinducers are the most explicit candidate signal molecules to indicate that population density is sufficient for adaptation and that it is reasonable to terminate adaptive proliferation.

In *Pba*, two quorum sensing systems have been described to date: (1) mediated by 6-oxo- and 8-oxo-AHL—the products of the *expI* gene and (2) mediated by autoinducer of the second type, AI-2, the product of the *luxS* gene [8]. AHL, which is used as quorum sensing signal, not only by *Pectobacterium* species, but also by species of some other genera, are widely shown to control virulence, motility, biofilm formation, and oxidative stress resistance in *Pectobacterium* species [9,10,11]. In turn, the role of AI-2, which is a “universal” inter-species signal produced by most, if not all, bacteria, in *Pectobacterium* physiological responses, is much less understood [11,12,13].

The aim of our study was to determine whether AHL and AI-2 are involved in the termination of adaptive proliferation in *Pba*. In addition, we sought to check whether, other than *Pba* bacterial species, both AHL-producing and AHL-non-producing were able to implement adaptive proliferation, and if so, whether the signaling background that primes the termination of adaptive proliferation is genus-specific or universal for different bacterial genera, enabling the collaborative regulation of this adaptive program in multispecies communities.

## 2. Results

### 2.1. Effect of Exogenous AHL on the Adaptive Proliferation of Pba

**Figure 1:**
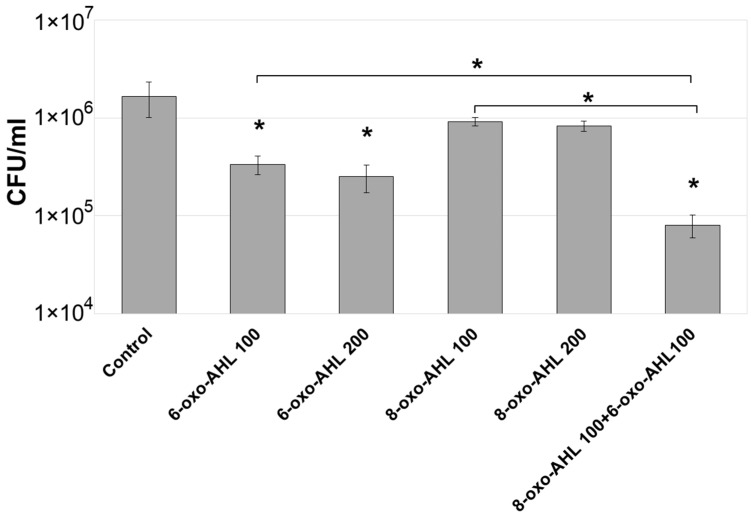
The colony forming unit (CFU) titer at which adaptive proliferation was terminated in *Pectobacterium atrosepticum* starving cultures with a low initial population density (10^3^ CFU/mL). Starving cultures were incubated in the absence of exogenous acyl homoserine lactones (AHL) (Control), or in the presence of either 100 or 200 µM of either 6-oxo-AHL or 8-oxo-AHL, or in the presence of both 6-oxo-AHL and 8-oxo-AHL each at 100 µM concentration. The presented values are the means ± SD of three biological replicates. Asterisks (*) show a significant difference (Mann–Whitney two-sided test, *p* < 0.05) from the control or between the variants designated by brackets.

Since the termination of adaptive proliferation is associated with the accumulation of AHL in the supernatants of starving cultures [6], we hypothesized that these autoinducers are those signals that stop cell division under starvation at low population density, and therefore, the exogenous application of 6-oxo-AHL and/or 8-oxo-AHL (AHL synthesized by *Pba*) can cause the premature termination of adaptive proliferation. To check this, starving cultures of low population density (10^3^ CFU/mL) were supplemented with 6-oxo-AHL and/or 8-oxo-AHL. Before this, we determined that, under growth-promoting conditions (in minimal growth medium D5), the concentration of AHL in *Pba* cultures reached 10.3 ± 2.3 µM. At this concentration, exogenous AHL did not affect adaptive proliferation, which was terminated at ~10^6^ CFU/mL, as well as in the starving cultures incubated without exogenous AHL. However, at the increased concentrations of 100 µM and 200 µM, exogenous 6-oxo-AHL led to the premature termination of adaptive proliferation, which occurred at CFU titers five- and seven-fold, respectively, lower than that in the cultures incubated without exogenous AHL (Figure 1). 8-oxo-AHL at 100 µM and 200 µM concentrations did not affect adaptive proliferation but enhanced the effect of 6-oxo-AHL: the termination of adaptive proliferation in the presence of both 6-oxo-AHL and 8-oxo-AHL (at 100 µM each) occurred at a cell titer that was 20-fold lower than that in the cultures incubated without exogenous AHL (Figure 1). Importantly, under growth-promoting conditions, cell proliferation was unaffected by the applied concentrations of AHL, indicating that the observed AHL-mediated premature termination of adaptive proliferation was not due to the growth-repressing effect of AHL. Thus, AHL can contribute to the termination of adaptive proliferation, but only at concentrations higher than those found in *Pba* cultures.

### 2.2. Adaptive Proliferation and Its Consequences in ΔexpI and ΔluxS Pba Mutants

**Figure 2:**
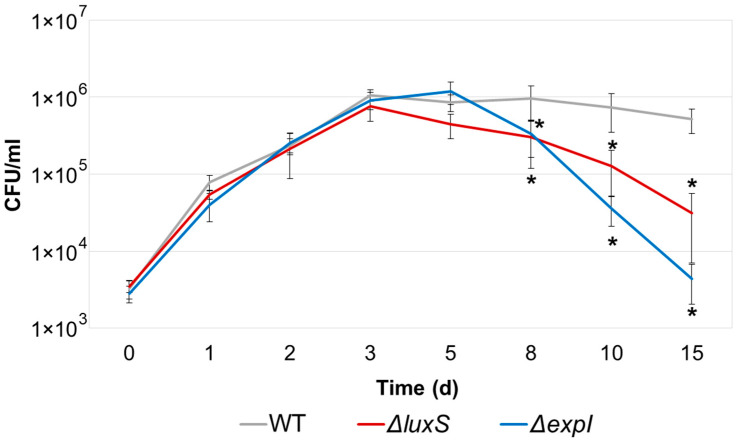
The dynamics of the colony forming unit (CFU) titer in the starving cultures of the wild type *Pectobacterium atrosepticum* (WT, grey line) and its Δ*expI* (blue line) and Δ*luxS* (red line) mutants. Cultures were incubated in a carbon-deficient medium at low initial population densities (2–4 × 10^3^ CFU/mL). The presented values are the means ± SD of four-five biological replicates. Asterisks (*) show a significant difference (Mann–Whitney two-sided test, *p* < 0.05) between the wild type and mutant strains at each time point.

To further investigate the role of intercellular communication in adaptive proliferation, we monitored the dynamics of CFU titer in the starving cultures with low initial population densities (2–3 × 10^3^ CFU/mL) of the wild type *Pba* and its quorum-deficient mutants with knocked out genes encoding AHL-synthase (*expI*) or AI-2-synthase (*luxS*). Adaptive proliferation proceeded in a similar way in the wild type and both mutants, and its termination occurred at a similar CFU level (~10^6^ CFU/mL) in all three strains (Figure 2). However, after the termination of adaptive proliferation (which occurred after three days of incubation), the CFU titer in the cultures of the wild type strain remained at a level of ~10^6^ CFU/mL up to 15 days of incubation, while in the cultures of both mutant strains, the CFU titer decreased (up to 5 × 10^4^ for Δ*luxS* mutant and 6 × 10^3^ for Δ*expI* mutant by the 15th day of incubation) (Figure 2). This means that the deficiency in either AHL or AI-2 does not affect adaptive proliferation and its termination at a population density of ~10^6^, but it causes a pronounced negative effect on the long-term fitness of *Pba* under starvation following adaptive proliferation. Therefore, we hypothesized that both AHL and AI-2 were required for *Pba* to acquire stress resistance during adaptive proliferation.

**Figure 3:**
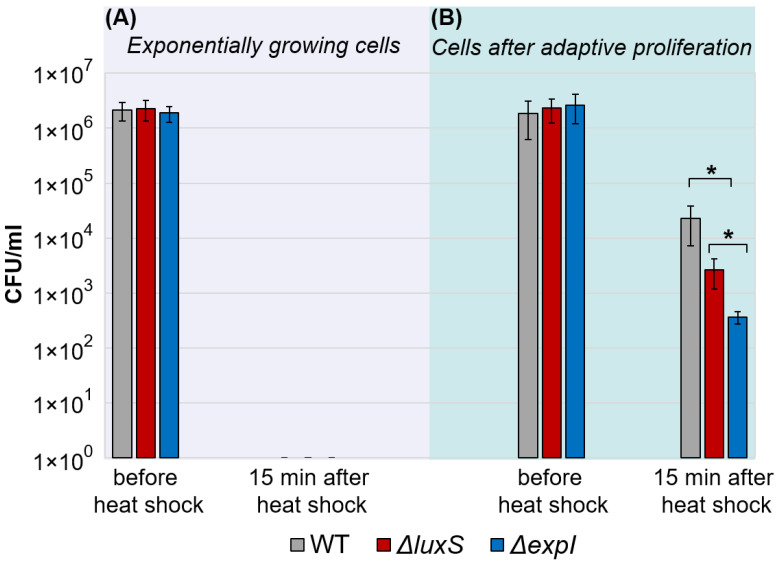
Heat shock resistance of the wild type *Pectobacterium atrosepticum* (WT, grey column) and its Δ*expI* (blue column) and Δ*luxS* (red column) mutants. Cells sustained under two culture conditions were assayed: (**A**) cells grown in LB medium until the late log phase and (**B**) cells that passed through adaptive proliferation in a carbon-deficient medium. The duration of the heat shock was 5 min. Fifteen minutes after the heat shock was abolished, and the cell suspensions were plated to determine the CFU titer. The presented values are the means ± SD of three biological replicates. Asterisks (*) show a significant difference (Mann–Whitney two-sided test, *p* < 0.05) between the variants designated by brackets.

To check this, for the wild type and both mutant strains, the resistance to heat shock (48 °C, 5 min) was compared for late exponential phase cells (cultured in LB medium) and starving cells that passed through adaptive proliferation in carbon-deficient medium. No CFU was recovered after heat shock from the suspensions of exponentially growing cells of all three strains (Figure 3A). In turn, in all three strains, some cells that passed through adaptive proliferation retained colony-forming ability after heat shock (Figure 3B). However, the proportion of such resistant cells differed in the three assayed strains: 1.2%, 0.11%, and 0.013% of CFUs recovered after heat shock in the suspensions of the wild type, Δ*luxS* mutant, and Δ*expI* mutant, respectively (Figure 3B). Thus, indeed, in the absence of either AHL or AI-2, *Pba* cells cannot acquire full (typical of the wild type) resistance following adaptive proliferation.

To test whether the AHL- or AI-2-deficiency affected not only stress resistance, but also the virulence of *Pba* cells following adaptive proliferation, tobacco plants were infected with late exponential phase or starving (after passing adaptive proliferation) cells of the wild type strain and its Δ*luxS* and Δ*expI* mutants. In total, 80%, 50%, and 10% of plants displayed disease symptoms following inoculation with exponentially growing cells of the wild type, Δ*luxS* mutant, and Δ*expI* mutant, respectively. In turn, 80%, 20%, and 0% showed disease development following inoculation with the starving cells of the wild type, Δ*luxS* mutant, and Δ*expI* mutant, respectively. Thus, in the case of the wild type, starving cells were as virulent as exponentially growing cells. In contrast, starving cells of the mutant strains were less virulent compared with the exponentially growing cells of the same strain. This means that both AHL- and AI-2 are required for *Pba* to maintain virulence following adaptive proliferation.

### 2.3. Effect of the Depletion of AHL or AI-2 on the Potential of Culture Supernatants to Cause the Premature Termination of Adaptive Proliferation

We have previously demonstrated that adaptive proliferation is terminated prematurely if *Pba* cells are incubated at a low population density not in a fresh carbon-deficient medium but in the supernatants of *Pba* starving cultures that had already passed through adaptive proliferation [6]. This suggests that, during adaptive proliferation, the metabolites that cause the termination of cell division are accumulated in cultural supernatants. Since knocking out the *expI* or *luxS* genes had no effect on the dynamics of adaptive proliferation or the CFU level at which it was terminated, it can be assumed that neither AHL nor AI-2 are those extracellular metabolites that contribute to the termination of adaptive proliferation. If this is true, then the supernatants of starving cultures (that passed through adaptive proliferation) of all three strains would have a similar effect in terms of causing the premature termination of adaptive proliferation. To check this, cells of the wild type strain, Δ*luxS* mutant, and Δ*expI* mutant were incubated at a low initial population density in a fresh carbon-deficient medium or in the supernatants of cultures that had passed through adaptive proliferation of either the wild type strain or Δ*luxS* mutant or Δ*expI* mutant. The supernatants of the wild type strain theoretically contained both AHL and AI-2, the supernatants of Δ*expI* lacked AHL but contained AI-2, and the supernatants of Δ*luxS* lacked AI-2 but contained AHL.

**Figure 4:**
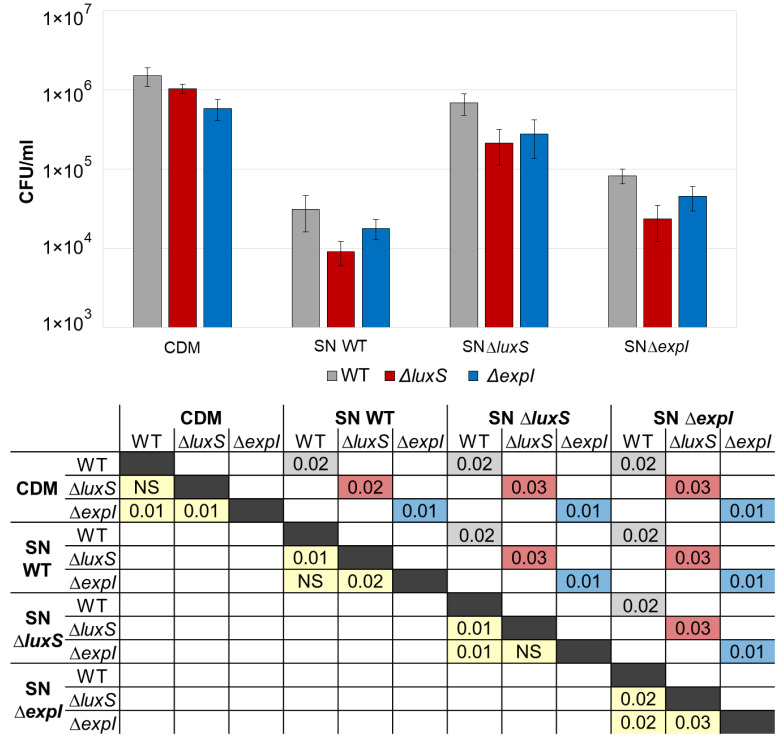
The effect of supernatants (SN) from the wild type *Pectobacterium atrosepticum* (SN WT), Δ*expI* mutant (SN Δ*expI*), and Δ*luxS* mutant (SN Δ*luxS*) starving cultures that passed through adaptive proliferation on the adaptive proliferation of the wild type (grey columns), Δ*expI* mutant (blue columns), and Δ*luxS* mutant (red columns). Cultures were incubated in fresh carbon-deficient medium (CDM) or supernatants of *P. atrosepticum* starving cultures at low initial population densities (2–4 × 10^3^ CFU/mL). The presented values are the means ± SD of three biological replicates. The table located under the diagram shows significant differences (*p*-values) between the designated experimental groups (Mann–Whitney two-sided test with Bonferroni correction for multiple comparison, *p* < 0.05). NS—non-significant.

The supernatants of the wild type strain caused the premature termination of adaptive proliferation at a level of 1–4 × 10^4^ CFU/mL, whereas, in a fresh medium, the termination of adaptive proliferation occurred at 9 × 10^5^–2 × 10^6^ CFU/mL (Figure 4). The supernatants of the Δ*luxS* mutant, lacking AI-2, also had a slight (but statistically significant) effect on causing the premature termination of adaptive proliferation, but this effect was much lower than that of the supernatants of the wild type (Figure 4). In the absence of AHL (supernatants of the Δ*expI* mutant), the CFU level at which the adaptive proliferation was terminated was lower than that in the supernatants of the Δ*luxS* mutant but higher than that in the supernatants of the wild type (Figure 4). Thus, our results show that both AHL and AI-2 participate in the termination of adaptive proliferation, with AI-2 contributing more to this process than AHL. However, when one of the two types of quorum molecules is excluded from the signaling pool, the potential of the supernatants to cause the premature termination of adaptive proliferation is not entirely eliminated but just diminished.

### 2.4. The Relative Levels of AHL and AI-2 in the Cultural Supernatants of the Wild Type Strain, ΔluxS and ΔexpI Mutants following Adaptive Proliferation

The obtained results showed that, on the one hand, both AHL and AI-2 contributed to the termination of adaptive proliferation (Figure 4), and, on the other hand, in AHL-deficient and AI-2-deficient strains, the termination of adaptive proliferation occurred at a similar CFU level as in the wild type strain, producing both autoinducers (Figure 2). This contradiction can be explained by the existence of compensatory mechanisms that can offset the effect of the absence of one of the autoinducers involved in the termination of adaptive proliferation. We, therefore, questioned whether the absence of one of the analyzed autoinducers influenced the level of the other one.

**Figure 5:**
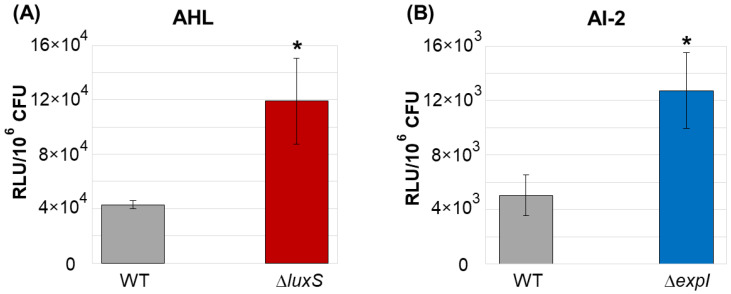
The levels of acyl homoserine lactones (AHL) (**A**) and autoinducer-2 (AI-2) (**B**) in the supernatants of starving cultures of *Pectobacterium atrosepticum* that passed through adaptive proliferation. AHL was measured in the wild type strain (WT, grey column) and AI-2-deficient Δ*luxS* mutant (Δ*luxS*, red column) (**A**); AI-2 was measured in the wild type strain (WT, grey column) and AHL-deficient Δ*expI* mutant (Δ*expI*, blue column) (**B**). The presented values are the means ± SD of three biological replicates. Asterisks (*) show a significant difference (Mann–Whitney two-sided test, *p* < 0.05).

To check this, we used bioluminescent reporter strains to monitor the relative content of AHL (strain *E. coli* JLD271 pAL103) and AI-2 (strain *Vibrio harveyi* BB170) in the supernatants of starving cultures of the wild type *Pba* and Δ*luxS* and Δ*expI* mutants. During the first two days of incubation in carbon-deficient medium at a low initial population density (2–4 × 10^3^ CFU/mL), the concentrations of AHL and AI-2 in all assayed starving cultures were below the level that the reporter strains could detect. By the third day of incubation, when the adaptive proliferation had terminated, the levels of autoinducers were detectable (with the exception of AHL in Δ*expI* mutant and AI-2 in Δ*luxS* mutant). The level of AHL was 2.8-times higher in the supernatants of the Δ*luxS* mutant than that of the wild type, while the level of AI-2 was 2.5-times higher in the supernatants of the Δ*expI* mutant than that of the wild type (Figure 5). Thus, the absence of one of the autoinducers leads to the increased accumulation of the other one during adaptive proliferation.

### 2.5. Effect of Supernatants of Pba Starving Cultures on the Adaptive Proliferation of Bacteria of Different Species

**Figure 6:**
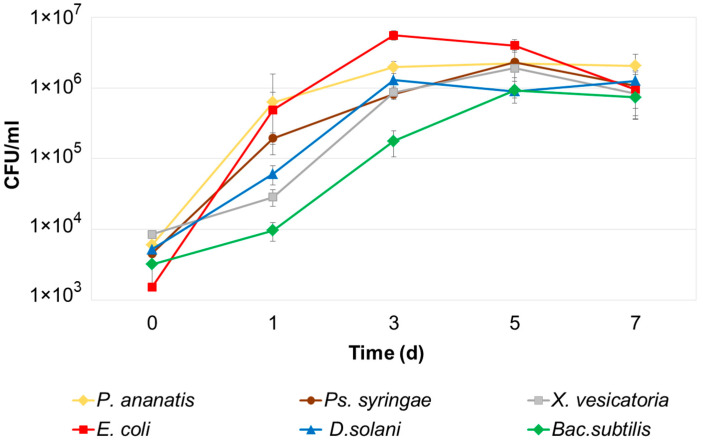
The dynamics of the colony forming unit (CFU) titer in the starving cultures of *Pantoea ananatis* (yellow line), *Pseudomonas syringae* (black line), *Xanthomonas vesicatoria* (grey line), *Escherichia coli* (red line), *Dickeya solani* (blue line), and *Bacillus subtilis* (green line). Cultures were incubated in a carbon-deficient medium at low initial population densities (2–9 × 10^3^ CFU/mL). The presented values are the means ± SD of three biological replicates.

Given our findings that AHL play a role in priming the termination of adaptive proliferation in *Pba*, we decided to assess the effect of *Pba* starving culture supernatants on the adaptive proliferation of other bacterial species, both producing and not producing AHL. Since adaptive proliferation has been previously described only for *Pba*, we first investigated whether, other than *Pba,* which bacterial species are able to implement this adaptive program. All six analyzed bacterial species (*Pantoea ananatis*, *Pseudomonas syringae*, *Xanthomonas vesicatoria*, *Dickeya solani*, *Escherichia coli*, and *Bacillus subtilis*) implemented adaptive proliferation when cells were transferred into a carbon-deficient medium at a low (2–9 × 10^3^ CFU/mL) initial cell density; the adaptive proliferation proceeded until cultures reached a population density of 1–6 × 10^6^ CFU/mL (Figure 6).

**Figure 7:**
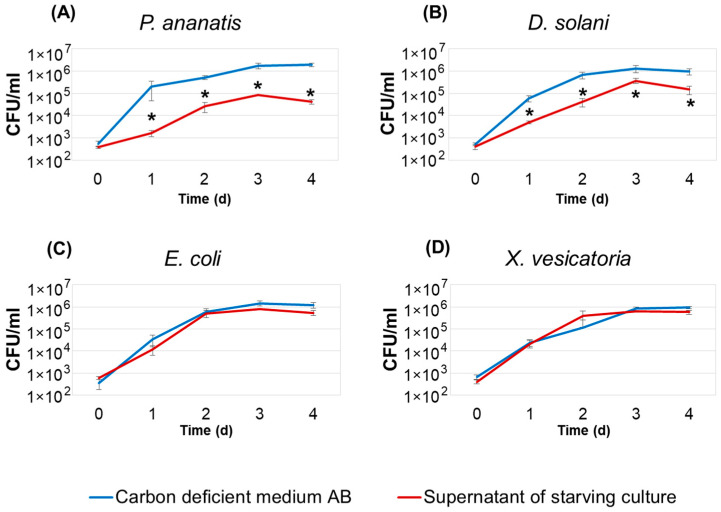
The effect of the supernatants of the *Pectobacterium atrosepticum* starving cultures that passed through adaptive proliferation on the adaptive proliferation of *Pantoea ananatis* (**A**), *Dickeya solani* (**B**), *Escherichia coli* (**C**), and *Xanthomonas vesicatoria* (**D**). Cultures were incubated at low initial population densities in either a fresh carbon-deficient medium (blue line) or supernatants of *P. atrosepticum* starving cultures (red line). The presented values are the means ± SD of three biological replicates. Asterisks (*) show a significant difference (Mann–Whitney two-sided test, *p* < 0.05) between the colony forming unit (CFU) titers in cultures incubated in a fresh carbon-deficient medium vs. that in cultures incubated in the supernatants of the *P. atrosepticum* starving cultures.

Then, the dynamics of adaptive proliferation in a fresh carbon-deficient medium and the supernatants of *Pba* starving cultures that passed through adaptive proliferation were compared for two AHL-producing (*P. ananatis* and *D. solani*) and two AHL-non-producing (*X. vesicatoria* and *E. coli*) species (Figure 7).

In *Pba* cultural supernatants, adaptive proliferation of *P. ananatis* proceeded slowly and terminated at a 20-times lower CFU level than in a fresh carbon-deficient medium (Figure 7A). *D. solani* also implemented adaptive proliferation more slowly and to a five-fold lower CFU level in *Pba* cultural supernatants than in a fresh carbon-deficient medium (Figure 7B). In contrast, adaptive proliferation of both AHL-non-producing species (*X. vesicatoria* and *E. coli*) proceeded in a similar way in *Pba* cultural supernatants and in a fresh carbon-deficient medium (Figure 7C,D). Thus, the extracellular metabolites of *Pba* starving cultures that have passed through adaptive proliferation contribute to the termination of adaptive proliferation in other AHL-producing species but not in AHL-non-producing species.

## 3. Discussion

In the present study, we aimed to understand for what reasons bacterial cell division under starvation at low initial population densities (adaptive proliferation) is terminated when the population density reaches a value of 10^6^ CFU/mL. We put forward the hypothesis that it is the accumulation of the quorum-related autoinducers that primes the termination of adaptive proliferation. We first examined whether exogenous AHL would repress the adaptive proliferation. We showed that exogenous 6-oxo-AHL caused premature termination of adaptive proliferation, whereas 8-oxo-AHL enhanced the effect of 6-oxo-AHL. However, this effect of AHL was manifested only at concentrations higher than those accumulated in *Pba* cultures.

Then, we monitored adaptive proliferation in two quorum-related *Pba* mutants, one of which with knocked out gene encoding AHL-synthase (Δ*expI*) was AHL-deficient, while the other one with knocked out gene encoding AI-2-synthase (Δ*luxS*) was AI-2-deficient. Unexpectedly, in both mutant strains, the adaptive proliferation proceeded similarly to the wild type strain, including that it was terminated at a population density of 10^6^ CFU/mL. However, in mutant strains, in contrast to the wild type strain, we observed a rather sharp decrease in CFU titers following adaptive proliferation. We proposed that both AHL- and AI-2-mediated quorum sensing systems were required to gain stress resistance following adaptive proliferation. Indeed, following adaptive proliferation, the level of cross-protection and the level of virulence were reduced in both mutants compared with the wild type.

Based on the monitoring of adaptive proliferation in the Δ*expI* and Δ*luxS* mutants, AHL or AI-2 were required for obtaining the increased stress resistance, but they were unlikely to be involved in priming the termination of adaptive proliferation. Then, we assumed that if these two autoinducers do not indeed participate in the target physiological process, then the supernatants of cultures of mutant strains that passed through adaptive proliferation would cause the premature termination of adaptive proliferation, similar to the supernatants of the wild type. However, compared to the supernatants of the wild type cultures, the supernatants from mutant strains had a reduced ability to prime the premature termination of adaptive proliferation, especially the supernatants of the Δ*luxS* mutant. This fact clearly shows that both AHL and AI-2 (especially the latter) are involved in priming the termination of adaptive proliferation. However, at the same time, it remained unclear why the termination of adaptive proliferation in AHL- and AI-2-deficient strains occurred in a manner similar to the wild type strain.

We proposed that the loss of one of the two studied autoinducers (AHL or AI-2) can be compensated for by the enhanced activity of the other or by additional yet unidentified intercellular signaling molecules, which would lead to the termination of adaptive proliferation in AHL- and AI-2-deficient strains. To check this, we determined the relative levels of AHL and AI-2 in the wild type and mutant strains following adaptive proliferation. Indeed, in the supernatants of starving cultures of the Δ*expI* mutant, we observed an increased level of AI-2 compared to the wild type, while in those of the Δ*luxS* mutant, an increased level of AHL accumulated. So, if a strain is unable to produce one quorum-related autoinducer during adaptive proliferation, it produces a greater amount of the other one, which presumably contributes to the termination of adaptive proliferation, even in the absence of one of the regulators of this process. This is in accordance with our results showing that only increased concentrations of exogenous AHL could trigger the premature termination of adaptive proliferation in *Pba*. Thus, the termination of adaptive proliferation is triggered by the combinatory and mutually compensatory actions of two quorum sensing systems and maybe other additional metabolites involved in intercellular communication that remain to be identified. Herewith, the interaction of different regulatory systems during the termination of adaptive proliferation is likely to be organized in such a way as to ensure the successful realization of this physiological process, even in the case of a failure of one of the regulatory systems involved in its regulation.

Quorum sensing autoinducer signal integration expressed in the cooperative action of two quorum sensing systems in terms of the activation of various phenotypes has been previously reported for *Pseudomonas aeruginosa* and the bioluminescent bacterium *Vibrio harveyi* [14,15,16]. In *V. harveyi*, different quorum-related phenotypes were manifested at different ratios of AI-2 and AHL concentrations. Herewith, the manifestation of some phenotypes required the presence of both autoinducers, while other phenotypes could be induced by only one of the autoinducers, and the second one only enhanced the effect of the first autoinducer [16]. Our results show that AI-2 and AHL seem to enhance each other’s actions in terms of priming the termination of adaptive proliferation, but neither of these two autoinducers is strictly required for such priming.

The results of the conducted experiments also indicate that the specific extracellular signaling background is not the sole element required for the termination of adaptive proliferation. Additionally, during adaptive proliferation, the sensitivity (competence) of cells to this extracellular signaling background must be increased. We were able to cause the premature termination of adaptive proliferation (at CFU levels below 10^6^ CFU/mL) using various treatments, but we were never able to completely repress adaptive proliferation, even when cells were transferred to supernatants of wild type cultures that had passed through adaptive proliferation, despite the fact that these supernatants contained the entire extracellular signaling background for the termination of adaptive proliferation. In our opinion, de novo inoculated cells are unable to perceive this signaling background and, due to this, proceed to the division. However, after several rounds of division, cells acquire the ability to recognize these signals, and adaptive proliferation is terminated prematurely. This scenario implies that bacterial cell sensitivity to quorum sensing signals is not constant and can change depending on the physiological status. Indeed, it has been previously shown that bacteria (*V. harveyi*) can adjust the sensitivity to quorum sensing signals [17,18]. In turn, such an adjustment of the sensitivity to autoinducers is likely to play an important role in the termination of adaptive proliferation.

In our study, we also found that the phenomenon of adaptive proliferation is not unique to *Pba* and assessed whether the signaling background that primes the termination of adaptive proliferation is genus-specific or common in different genera. All six analyzed bacterial species were able to implement adaptive proliferation during starvation at a low population density. The termination of adaptive proliferation in different species occurred at 1–6 × 10^6^ CFU/mL. The supernatants of starving *Pba* cultures could cause premature termination of adaptive proliferation in other AHL-producing species, but not in AHL-non-producing species. This fact additionally supports the role of AHL in the termination of adaptive proliferation in AHL-producing species. However, within AHL-producing species, the composition of the signaling background that primes the termination of adaptive proliferation seems to have some genus-specific features, since supernatants of *Pba* had a greater inhibitory effect on the adaptive proliferation of *P. ananatis* than of *D. solani*. The reason for this may be that the *Dickeya* species, in addition to producing AHL, synthesize another autoinducer, vfm (virulence factor modulating), as the main quorum sensing signal [19,20].

It has been previously shown that bacteria within complex communities perceive not only self-produced quorum-related molecules, but also those that are produced by other species [21,22,23]. This is quite expected for AI-2, which is synthesized by most bacterial species. Herewith, AHL also widely participated in interspecies communication [24,25,26,27]. Due to this, the bacteria of a particular species can sense not only their own population density, but also the density of the whole bacterial community (or its significant part) and exploit autoinducers produced by other members of the community. For example, *Pectobacterium wassabiae* (a phytopathogenic bacterium closely related to *Pba*) can sense AHL synthesized by other bacteria in the multispecies community of potato tubers, and an integral AHL pool produced by the whole community determines the level of virulence of this species [26]. Our results show that the composition of an integral pool of quorum-related molecules determined by the composition of the bacterial community can influence not only virulence, but also adaptive reactions in bacteria. Herewith, bacteria of different species can use quorum-related molecules of each other to initiate the adaptive program. In particular, adaptive proliferation is likely to be regulated at an interspecies level in multispecies communities.

Thus, our study shows that adaptive proliferation is a widespread phenomenon in the bacterial world. The existence of this phenomenon indicates that bacterial cells contain a significant amount of resources and energy that can be spent to provide multiple cell divisions in the absence of a growth substrate in order to successfully adapt to unfavorable conditions. The consumption of these resources is tightly regulated via intercellular communication to avoid their waste when the quorum level of population density required for adaptation is achieved. Our study provides the first information about the components of the extracellular signaling background that prime the termination of adaptive proliferation. These components are AHL and AI-2, which act synergistically and mutually compensatory (possibly with other as-yet unidentified metabolites) to provide timely termination of adaptive proliferation.

## 4. Materials and Methods

### 4.1. Bacterial Strains, Media and Culture Conditions

*Pectobacterium atrosepticum* SCRI1043 (*Pba*) (ATCC BAA-672), *Pantoea ananatis* LMG20103, *Dickeya solani* DSM28711, *Xanthomonas vesicatoria* DSM22252, *Escherichia coli* K-12 MG1655, *Pseudomonas syringae* DC3000, and *Bacillus subtilis* DSM10 were routinely grown in Luria-Bertani (LB) medium on a rotary shaker (180 rpm) at 28 °C. The *Pba* Δ*expI* and Δ*luxS* mutant strains were grown under similar conditions in the presence of kanamycin (20 μg/mL). *E. coli* JLD271 was grown at 37 °C in the presence of tetracycline (20 mg/mL). *Vibrio harveyi* BB170 and BB152 strains were grown in LB medium, containing 20.0 g/L NaCl at 28 °C. To determine the level of AHL in the *Pba* cultures under growth-promoting conditions, bacteria were cultured on a rotary shaker (180 rpm) at 28 °C in the synthetic medium D5, containing 1.0 g/L NH_4_Cl, 0.1 mM Na-K phosphate buffer (pH 7.5), 0.3 g/L MgSO_4_ × 7H_2_O, and 2.0 g/L sucrose.

To obtain starving cultures of the studied bacteria, cells grown in LB medium were harvested (8000× *g*, 20 °C, 10 min) at the late logarithmic growth phase (~1–2 × 10^9^ CFU/mL) and washed twice in a mineral carbon-free AB medium, which contained 1.0 g/L NH_4_Cl, 0.62 g/L MgSO_4_ × 7H_2_O, 0.15 g/L KCl, 0.013 g/L CaCl_2_ × 2H_2_O, pH 7.5. The cells were then resuspended in AB medium, and 10-fold dilutions were prepared. The resultant starving cultures, with initial population densities of 10^2^–10^4^ CFU/mL, were incubated in glass vials without aeration at 28 °C. To assess the effect of AHL on adaptive proliferation, starving cultures were supplemented with different concentrations of N-(3-oxohexanoyl)-L-homoserine lactone and/or N-(3-oxooctanoyl)-L-homoserine lactone (Santa Cruz Biotechnology, Inc., Dallas, TX, USA).

To obtain the supernatants of cultures that passed through adaptive proliferation, starving cultures with low initial population densities (10^2^–10^4^ CFU/mL) were incubated for four days. After that, first a bulk of cells was harvested (10,000× *g*, 20 °C, 10 min), and then the supernatants were filtered through nitrocellulose filters (0.2 μm) (Corning, Berlin, Germany) under sterile conditions. The absence of bacteria in the supernatants was confirmed by plating on 1.5% LB-agar. The obtained supernatants were inoculated with 5 × 10^2^–4 × 10^3^ CFU/mL of the exponentially growing cells of either *Pba*, or *Pantoea ananatis*, or *Dickeya solani*, or *Escherichia coli*, or *Xanthomonas vesicatoria*, and the cell titers in the obtained cell suspensions were monitored by plating on 1.5% LB-agar.

### 4.2. Construction of expI and luxS Deletion Mutants

The *expI* and *luxS* deletion mutants (Δ*expI* and Δ*luxS*) were constructed by the method described by Kaniga et al. [28]. Since the 3’-end of the *expI* gene in the *Pba* genome overlapped with the 3’-end of the *expR* gene (16 bp) located on the complementary DNA strand, during mutagenesis, we removed only that part of the *expI* gene that did not overlap with the *expR* gene. The target gene *expI* (ECA0105 locus), together with the adjacent regions (approximately 1000 bp up- and downstream of *expI* ORF), were amplified by PCR with primers up*expI*F and dn*expI*R (Table S1) using Q5 high-fidelity DNA polymerase (NEB, Ipswich, MA, USA). The amplified PCR fragment was cloned into the bacterial cloning vector system pGEM-T Easy (Promega, Madison, WI, USA). The obtained plasmid (pGEM:*expI*) was introduced into *E. coli* NovaBlue by chemical transformation. Transformants carrying the recombinant plasmid were screened by ampicillin resistance and further verified by PCR using plasmid specific primers for the T7 and SP6 polymerase promoters, which flank the multiple cloning regions of pGEM-T Easy.

To replace *expI* ORF with the Km^R^ cassette, a part of the pGEM:*expI* plasmid (including ~1000 bp regions up- and downstream of *expI* ORF but not *expI* ORF itself) was amplified with primers dn*expI*KmF and up*expI*KmR (Table S1), whose 5′-ends were complementary to the end regions of the Km^R^ cassette. The amplified PCR fragment was treated with restriction endonuclease DpnI to remove the original methylated plasmid and then purified using a DNA cleanup kit (NEB, Ipswich, MA, USA). The Km^R^ cassette was amplified from the pKD4 plasmid with primers Km*expI*F and Km*expI*R, whose 5′-ends were complementary to *Pba* DNA regions adjacent to *expI* ORF. Two obtained PCR fragments (corresponding to pGEM plasmid with ~1000 bp regions up- and downstream of *expI* ORF and to Km^R^ cassette) were joined by the circular polymerase extension cloning method [29]. The obtained plasmid (pGEM:Δ*expI*;Km^R^) was introduced into *E. coli* NovaBlue by chemical transformation. The mutant locus was confirmed by DNA sequencing.

The mutant locus (containing the Km^R^ cassette and ~1000 bp regions up- and downstream of *expI* ORF) was amplified with primers up*expI*F and dn*expI*R and ligated (T4 ligase, NEB, Ipswich, MA, USA) into the SmaI-digested (NEB, Ipswich, MA, USA) suicide vector pKNG101 to generate the recombinant plasmid containing the allelic exchange cassette for the target locus. The obtained plasmid (pKNG101:Δ*expI*;Km^R^) was introduced into *E. coli* cc118 by electroporation. The transfer of pKNG101:Δ*expI*;Km^R^ plasmid from *E. coli* cc118 into *Pba* was achieved by triparental mating using *E. coli* HH26 as a helper strain. The clones, in which pKNG101:Δ*expI*;Km^R^ plasmid was integrated into the chromosome by a single crossover event, were selected by streptomycin and kanamycin resistance. The clones, in which the second crossover event led to the replacement of the target locus with the mutant one and the donor plasmid was eliminated, were selected on M9 agar medium containing 10% sucrose. Then, the clones were tested for sensitivity to streptomycin. Clones without streptomycin resistance were analyzed by PCR with primers Check*expI*F and Check*expI*R to identify Δ*expI* mutants. The Δ*luxS* (ECA3362 locus) mutant was constructed in a similar manner using the primers given in the Table S1.

### 4.3. Stress Tolerance Assay

To compare the stress resistance of the late exponential phase and starving (after passing through the adaptive proliferation) cells of the wild type *Pba* and its Δ*expI* and Δ*luxS* mutants, cells were exposed to a heat challenge. Late exponential phase cells were cultured in LB medium, and starving cells were obtained by incubating under starvation conditions at a low initial population density (~5 × 10^3^) for four days (during which the adaptive proliferation had passed). Late exponential phase and starving cells were harvested and resuspended in a carbon-deficient AB medium up to a density of ~10^6^ CFU/mL. For the heat challenge, 100 µL aliquots of cell suspensions were exposed to 48 °C for 5 min. Fifteen min after heat shock, suspensions were plated onto 1.5% LB agar as serial 10-fold dilutions. The plates were incubated at 28 °C for two days before the CFUs were counted.

### 4.4. Virulence Assay

The virulence of the late exponential phase and starving (after passing through the adaptive proliferation) cells of the wild type *Pba* and its Δ*expI* and Δ*luxS* mutants was compared using *Nicotiana tabacum* cv. Petit Havana SR1 plants. Plants were grown axenically in test tubes placed in a growth chamber with a 16-h light/8-h dark cycle photoperiod. Seeds were surface-sterilized using diluted bleach (0.8% of active chlorine) and 1% sodium dodecyl sulfate for 30 min, washed seven times with sterile distilled water, and then transferred to Murashige and Skoog medium (MS) in Petri dishes. Ten-day-old seedlings were transferred to individual flasks containing MS. Five weeks after planting, the plants were infected with bacteria. Two types of inoculums were used. First, bacteria were grown until the late logarithmic phase (~1 × 10^9^ CFU/mL) in LB medium, washed with sterile 10 mM MgSO_4_, and resuspended in 10 mM MgSO_4_ up to a density of ~1 × 10^7^ CFU mL^−1^. Second, bacteria were incubated under starvation conditions at a low initial population density (~5 × 10^3^) for four days (during which the adaptive proliferation had passed), and then cells were harvested and resuspended up to ~1 × 10^7^ CFU mL^−1^ in sterile 10 mM MgSO_4_. Then, sterile 10 mM MgSO_4_ (control plants) or bacterial suspensions containing ~1 × 10^5^ cells (late log or starving) were placed as 10 μL drops into the bosoms of the leaves in the middle part of the stems using sterile pipette tips, and slight scratches were made simultaneously. Symptoms were scored three days after plant inoculation.

### 4.5. Extraction and Detection of AHL and AI-2

To assess the AHL level, cultures were centrifuged for 10 min at 24,000× *g* at 4 °C, and the supernatants were filtered through nitrocellulose filters (0.2 μm) (Corning, Germany). AHLs were extracted from the obtained cell-free supernatants with an equal volume of ethyl acetate (acidified with 0.1 mL/L glacial acetic acid) for 4 h on a rotating mixer. The samples were centrifuged for 5 min at 9000× *g* at 4 °C, and the ethyl acetate phase was collected. The ethyl acetate was removed by rotary evaporation, and the residual ethyl acetate was evaporated under nitrogen gas [30]. The samples were diluted in 100 µL of water and used for the AHL bioassay. The level of AHL was analyzed using *E. coli* JLD271, carrying the bioluminescence reporter vector pAL103 or the vector pAL104 as a negative control [31]. The reporter strain was grown as described above, diluted up to an OD_600_ of 0.5, and the obtained suspension was transferred to a 96-well microtiter plate (180 µL per well). Twenty µL of water-dissolved ethyl acetate extracts of the supernatants of the tested cultures were added to the wells with *E. coli* JLD271. The plate was incubated for 3 h at 37 °C, and the luminescence from each well was detected using the microplate reader CLARIOstar (BMG Labtech GmbH, Ortenberg, Germany). Commercial N-(3-oxohexanoyl)-L-homoserine lactone (Sigma) was used as a control.

AI-2 in *Pba* cultural supernatants was determined by the bioluminescent method [32]. *Vibrio harveyi* strains BB170 (luxN::Tn5, sensor AI-2) and BB152 (luxI::Tn5, AI-2 producer) were kindly provided by Dr. B.L. Bassler. *V. harveyi* cultures were grown overnight as described above, and then the cells were diluted by 1:2000 in the medium, containing 17.5 g/L NaCl, 12.3 g/L MgSO_4_, 2.0 g/L casamino acids, 10 mM KH_2_PO_4_, 1 mM arginine, 1% glycerol, and pH 7.5. An amount of 180 µL aliquots of the reporter strain were added to the wells of a 96-well microtiter plate, and then 20 µL of supernatants from the tested cultures were added to the wells. The plate was incubated for 5 h at 28 °C. The supernatant from *V. harveyi* BB152 and the sterile medium were used as positive and negative controls, respectively [33]. The luminescence from each well was detected using the microplate reader CLARIOstar (BMG Labtech GmbH, Ortenberg, Germany).

### 4.6. Statistical Analysis

Statistical analysis was performed in OriginPro 9.8.0.200 using the Mann–Whitney two-sided test. Each experiment was carried out at least in triplicate. For multiple comparisons, a Mann–Whitney two-sided test with Bonferroni correction was used. The value of *p* < 0.05 was considered significant.

## 5. Conclusions

Both known quorum sensing mediators of *Pba* (AHL and AI-2) are components of the signaling background that “notify” *Pba* that it is time to terminate adaptive proliferation and proceed to the persistence-related stage of adaptation. AI-2 seems to have a greater contribution to the termination of adaptive proliferation than AHL. However, neither AI-2 nor AHL are strictly required to terminate adaptive proliferation, and these two signaling molecules can compensate for each other’s actions (at least partially) during the termination of adaptive proliferation, possibly along with some other as-yet unidentified signaling molecules. In turn, both AHL and AI-2 are required for the formation of cross-resistance in the course of adaptive proliferation and for retaining full virulence following adaptive proliferation; herewith, the AHL contributes more to cross-resistance than AI-2. Adaptive proliferation is a phenomenon common to a wide range of bacterial species that use different molecules as quorum sensing signals. The signaling background that primes the termination of adaptive proliferation in one AHL-producing species can be perceived by and terminate adaptive proliferation in (at least partially) other AHL-producing species, but not in AHL-non-producing species. This indicates that adaptive proliferation can be regulated at the interspecies level in complex bacterial communities. Bacterial cells “prepare” themselves for perceiving the signaling background that primes the termination of adaptive proliferation; the sensitivity to this signaling background is absent (or low) in exponentially growing cells, but it gradually develops during the course of adaptive proliferation.

## Data Availability

Not applicable.

## Supplementary Materials

The following supporting information can be downloaded at: https://www.mdpi.com/article/10.3390/ijms24087266/s1. References [34,35,36,37,38,39,40,41,42] are cited in the supplementary materials.
