# Supplementary material for: The Role of Intercellular Signaling in the Regulation of Bacterial Adaptive Proliferation
Source: Int J Mol Sci. 2023 Apr 14;24(8):7266. doi: 10.3390/ijms24087266 (PMC10138535; doi:10.3390/ijms24087266)
Supplement: Supplementary file 1 [file ijms-24-07266-s001.zip › ijms-2289298-supplementary.pdf]

Table S1. Bacterial strains, plasmids and primers used in this study.

**Name**	**Description**	**Origin**
**Strain**		
Pectobacterium atrosepticum SCRI1043 (*Pba*)	Wild type	[1]
Pectobacterium atrosepticum SCRI1043*ΔexpI*	Mutant strain of SCRI1043 containing the kanamycin cassette in the chromosome; *Km^R*	This study
Pectobacterium atrosepticum SCRI1043*ΔluxS*	Mutant strain of SCRI1043 containing the kanamycin cassette in the chromosome; *Km^R*	This study
Escherichia coli cc118	Host for suicidal vector pKNG101; *Δ(ara, leu) araD ΔlacX 74 galE galK PhoA20 thi-1 rpsE rpoB argE (am) recA1, Sm^R*	[2]
Escherichia coli HH26/pNJ5000	Mobilizing strain for conjugative transfer of the suicide vector pKNG101 into *Pba* cells; *tra+; Tet^R*	[3]
Escherichia coli NovaBlue	*endA 1hsdR17 (rK12–mK12+) supE44 thi-1 recA1 gyrA96 relA1 lacF′ [proA+ B+ lacIqZ ΔM15::Tn10 (Tet^R)]*	Novagen
Pantoea ananatis LMG 20103	Wild type	[4]
Dickeya solani DSM28711	Wild type	[5]
Xanthomonas vesicatoria DSM 22252	Wild type	[6]
Escherichia coli K-12 MG1655	Wild type	NCBI:txid511145
Pseudomonas syringae DC3000	Wild type	[7]
Bacillus subtilis DSM10	Wild type	[8]
E. coli JLD271	Host for the bioluminescence reporter vector pAL103 or pAL104 as a negative control; *WM54 sdiA271::cam*	[9]
Vibrio harveyi BB170	Strain was used as a sensor AI-2; *luxN::Tn5*	[10]
Vibrio harveyi BB152	Strain was used as a positive control; *luxI::Tn5*	[10]
**Plasmids**		
pGEM-T Easy	Linearized vector for cloning; *f1 ori Amp^R lacZ*	Promega
pGEM:*expI*	*f1 ori Amp^R lacZ expI*	This study
pGEM:*luxS*	*f1 ori Amp^R lacZ luxS*	This study
pGEM:*ΔexpI*;Km^R	*f1 ori Amp^R lacZ Km^R*	This study
pGEM:*ΔluxS*;Km^R	*f1 ori Amp^R lacZ Km^R*	This study
pKD4	Matrix for PCR amplification of kanamycin resistance cassette; *oriRγ rgnB bla Km^R*	[11]
pKNG101	Suicide mobilized vector for inactivation of target genes; *pir-ori R6K mobRK2 sacB Sm^R*	[12]
pKNG101:*ΔexpI*;Km^R	Suicide plasmid carrying mutant locus *ΔexpI*;Km^R; *Km^R Sm^R sacB*	This study
pKNG101:*ΔluxS*;Km^R	Suicide plasmid carrying mutant locus *ΔluxS*;Km^R; *Km^R SmR sacB*	This study
pAL103	The bioluminescence reporter vector; Tet^R; *luxR+luxI::luxCDABE; Tet^R p15A origin*	[9]
pAL104	The negative control for vector pAL103; Tet^R; *luxI::luxCDABE; Tet^R p15A origin*	[9]

Primers	
**Primer name**	**Primer sequence 5'—3'**
**Primers for mutagenesis** *ΔexpI*	
**up***expI*F	CGGCCGTCAGGAGATGTCTGAG
**dn***expI*R	ACAATTAGACTTAGGTACAGGTGTGG
**dn***expI*KmF	GCAGCTCCAGCCTACACGGAATTAGCGTAGTTGAACAAGG
**up***expI*KmR	CCCATGTCAGCCGTGTCAGCAAGAGTTCACACACG
**Km***expI*F	CGGCTGACATGGGGTGTAGGCTGGAGCTGCTTC
**Km***expI*R	GTGTAGGCTGGAGCTGCACGGCTGACATGGGAATTAG
**Check***expI*F	CCTGCTGACCAATGCTGAACTG
**Check***expI*R	ACATTCCCTGATGTTAGCCAATC
**Primers for mutagenesis** *ΔluxS*	
**up***luxS*F	CCTGCCTTATGCGACCTCTCTAC
**dn***luxS*R	CGCCTACACACTCAGCCTCG
**dn***luxS*KmF	CCATGTCAGCCGTTAAGACATATCTAGTTTATTGG
**up***luxS*KmR	CTCCAGCCTACACAATCAGTAACGGCATAATGCCTCC
**Km***luxS*F	CATTATGCCGTTACTGATTGTGTAGGCTGGAGCTGCTTC
**Km***luxS*R	CAATAAACTAGATATGTCTTAACGGCTGACATGGGAATTAGC
**Check***luxS*F	GCAGAAAGCGGGAAAAAGGC
**Check***luxS*R	CGCTGCTGACGGAGAACTGTTG

1. Bell, K.S.; Sebaihia, M.; Pritchard, L.; Holden, M.T.G.; Hyman, L.J.; Holeva, M.C.; Thomson, N.R.; Bentley, S.D.; Churcher, L.J.C.; Mungall, K.; et al. Genome sequence of the enterobacterial phytopathogen *Erwinia carotovora* subsp. *atroseptica* and characterization of virulence factors. *Proc. Natl. Acad. Sci.* **2004**, *101*, 11105–11110.
2. Herrero, M.; de Lorenzo, V.; Timmis, K.N. Transposon vectors containing non-antibiotic resistance selection markers for cloning and stable chromosomal insertion of foreign genes in gram-negative bacteria. *J. Bacteriol.* **1990**, *172*, 6557–6567.
3. Grinter, N.J. A broad-host-range cloning vector transposable to various replicons. *Gene* **1983**, *21*, 133–143.
4. De Maayer, P.; Chan, W.Y.; Venter, S.N.; Toth, I.K.; Birch, P.R.J.; Joubert, F.; Coutinho, T.A. The genome sequence of *Pantoea ananatis* LMG20103, the causative agent of *Eucalyptus* blight and dieback. *J. Bacteriol.* **2010**, 192, 2936–2937.
5. Van der Wolf, J. M.; Nijhuis, E. H.; Kowalewska, M. J.; Saddler, G. S.; Parkinson, N.; Elphinstone, J. G.; Pritchard, L.; Toth, I. K.; Lojkowska, E.; Potrykus, M.; Waleron, M.; de Vos, P.; Cleenwerck, I.; Pirhonen, M.; Garlant, L.; Hélias, V.; Pothier, J. F.; Pflüger, V.; Duffy, B.; Tsror, L.; Manulis, S. Dickeya solani sp. nov., a pectinolytic plant-pathogenic bacterium isolated from potato (Solanum tuberosum). Int J Syst Evol Microbiol. 2014, 64, 768-774.
6. Vauterin, L.; Hoste, B.; Kersters, K.; Swings, J. Reclassification of *Xanthomonas*. *Int.J.Syst.Bacteriol*. **1995**, *45*, 472-489.
7. Buell, C.R.; Joardar, V.; Lindeberg, M.; Selengut, J.; Paulsen, I.T.; Gwinn, M.L.; Dodson, R.J.; Deboy, R.T.; Durkin, A.S.; Kolonay, J.F.; Madupu, R.; Daugherty, S.; Brinkac, L.; Beanan, M.J.; Haft, D.H.; Nelson, W.C.; Davidsen, T.; Zafar, N.; Zhou, L.; Liu, J.; Yuan, Q.; Khouri, H.; Fedorova, N.; Tran, B.; Russell, D.; Berry, K.; Utterback, T.; Van Aken, S.E.; Feldblyum, T.V.; D'Ascenzo, M.; Deng, W.L.; Ramos, A.R.; Alfano, J.R.; Cartinhour, S.; Chatterjee, A.K.; Delaney, T.P.; Lazarowitz, S.G.; Martin, G.B.; Schneider, D.J.; Tang, X.; Bender, C.L.; White, O.; Fraser, C.M.; Collmer, A. The complete genome sequence of the *Arabidopsis* and tomato pathogen *Pseudomonas syringae* pv. tomato DC3000. *Proc. Natl. Acad. Sci. USA.* **2003**, *100*, 10181-10186.
8. Ansorg, R.; Zappel, H.; Thomssen, R. Bedeutung des Nachweises antibakterieller Stoffe im Urin für die bakteriologische Diagnostik und die Kontrolle der Chemotherapie von Harnwegsinfektionen. *Zentralbl.Bakteriol.Parasitenkd.Orig.Reihe*. **1975**, *230*, 492-507.
9. Lindsay, A.; Ahmer, B.M. Effect of *sdiA* on biosensor of N-acyl homoserine lactones. *J. Bacteriol*. **2005**, *187*, 5054–5058.
10. Surette, M.G.; Bassler, B.L. Quorum sensing in *Escherichia coli* and *Salmonella typhimurium*. *PNAS*. **1998**, *95*, 7046-7050.
11. Datsenko, K.A.; Wanner, B.L. One-step inactivation of chromosomal genes in *Escherichia coli* K-12 using PCR products. *Proc. Natl. Acad. Sci.* **2000**, *97*, 6640–6645.
12. Kaniga, K.; Delor, I.; Cornelis, G.R. A wide-host-range suicide vector for improving reverse genetics in gram-negative bacteria: Inactivation of the *blaA* gene of *Yersinia enterocolitica*. *Gene* **1991**, *109*, 137–141.
